# HIV Knowledge and Its Associated Sociodemographic Factors among Female Sex Workers in Malaysia

**DOI:** 10.21315/mjms2024.31.3.12

**Published:** 2024-06-27

**Authors:** Yong Kang Cheah, Anita Suleiman, Mazliza Ramly

**Affiliations:** 1 School of Economics, Finance and Banking, College of Business, Universiti Utara Malaysia, Kedah, Malaysia; 2 HIV/STI/Hepatitis C Sector, Ministry of Health Malaysia, Putrajaya, Malaysia

**Keywords:** female sex worker, HIV, knowledge, sexually transmitted infection, sociodemographic factors

## Abstract

**Background:**

Female sex workers (FSWs) have a high risk of human immunodeficiency virus (HIV) infection. In spite of the alarming fact that a large proportion of FSWs does not have adequate HIV knowledge, the association between sociodemographic factors and HIV knowledge among FSWs have yet to be thoroughly explored in the context of Malaysia. The aims of this study are the following: i) to determine HIV knowledge and ii) to examine the associated factors of HIV knowledge.

**Methods:**

An observational cross-sectional study was conducted. Data from the Integrated Biological and Behavioral Surveillance Survey (IBBS) 2017 (*n* = 630) were used. The survey was carried out in all states in Malaysia and its duration was 4 months (from March 2017 to June 2017). Ordered probit regressions were utilised to shed light on the association between sociodemographic variables and levels of HIV knowledge.

**Results:**

A large proportion of FSWs had middle-level HIV knowledge (44.1%). FSWs with tertiary-level education were 19.5% more likely to have high-level HIV knowledge compared to those without formal education. The probability of having low-level HIV knowledge was 6.8% lower among FSWs with monthly incomes of RM1,500–RM1,999 than those having incomes of ≤ RM499. Being single instead of married was associated with 7.6%–8% lower probabilities of having low- and middle-level HIV knowledge.

**Conclusion:**

Public health interventions to improve FSWs’ HIV knowledge need to take into consideration the role of sociodemographic factors.

## Introduction

Increases in numbers of people living with the human immunodeficiency virus (HIV) are considered a serious public health challenge across the globe. The alarming facts and figures show that nearly 2 million people were diagnosed with HIV in 2019 and up to 700,000 passed away because of HIV-related diseases (1). Worse still, only 30% of the total population was found to have comprehensive HIV knowledge (2). The latest report of the Ministry of Health Malaysia indicates that 92,063 Malaysians lived with HIV in 2020 and approximately 3,146 were new cases, which was equivalent to 9.3 cases per 100,000 population (3). It is widely evident that the HIV epidemic is dominated by people who inject drugs (PWIDs), female sex workers (FSWs), transgenders (TGs) and men who have sex with men (MSM) (4).

FSWs are the population with the highest risk of infection with HIV (5). Research shows that FSWs are 30 times more likely to acquire HIV compared with the general female population (6). There is also strong evidence suggesting that the prevalence of HIV among FSWs is approximately 20-fold higher than that of the general population (7, 8). The interaction between the general population and FSWs is unlimited as FSWs are available at public places. Therefore, FSWs play the main role in transmitting HIV to the public. They are often infected by their clients and eventually pass on to their sex partners. Moreover, FSWs tend to have poor socioeconomic status and educational backgrounds. Thus, their knowledge of preventing HIV is likely to be inadequate (9). Although FSWs are the priority population for HIV prevention measures, they are hard to reach and are not easily identified because sex work is illegal in Malaysia (10).

Apart from having high numbers of sexual partners, a workplace environment that does not allow FSWs to request the use of condoms and the law toward criminalisation of sex work are the major reasons for HIV infection among FSWs (7, 8). Numerous interventions directed toward reducing HIV transmission among FSWs have been suggested. These include condom utilisation, use of sexually transmitted infection (STI) diagnostic tests and peer support (11). Although these strategies are effective and may yield promising outcomes, the lack of HIV knowledge remains a challenge. The fact of the matter is that FSWs are unlikely to take any preventive measures if they do not have adequate HIV knowledge (12). As pointed out by Mutagoma et al. (13), high levels of HIV knowledge are the most basic method to reduce HIV infection. According to knowledge, attitude and practice (KAP), that is, a theory that focuses on what is known, believed and done related to health behaviours, adequate knowledge regarding HIV can encourage safe sex practice, thereby reducing the risk of HIV transmission (14). Therefore, policymakers should devote their attention to improving HIV knowledge among FSWs if the goal of reducing the prevalence of newly diagnosed HIV infection is to be achieved.

In Malaysia, owing to the laws that prohibit sex work, FSWs are marginalised communities and are often discriminated, maltreated and suffering from sexual violence (10). Therefore, more studies on HIV-related behaviours among FSWs are needed to formulate an effective, multidimensional HIV prevention intervention. The study by Wang et al. (8) was notable in investigating associated factors of HIV infection with a focus on FSWs in Malaysia. The authors found the use of illicit drugs to be a predictor of HIV infection, yet little is known regarding HIV knowledge among FSWs. The recent national HIV surveillance survey showed that approximately 41% of FSWs had adequate HIV knowledge in 2017 but it did not reveal detailed information on the association between sociodemographic factors and HIV knowledge (4).

Previous worldwide studies often concentrated on the prevalence of HIV knowledge among FSWs in China, Gambia, Peru, Rwanda and Philippines but did not examine the relationships between sociodemographic factors and HIV knowledge (7, 15–19). To the best of our knowledge, the present study is the first study that appears in literature that explores sociodemographic factors associated with FSWs’ HIV knowledge with concentration on Malaysia, an Islamic country, where Syariah law prohibits sex work, HIV is a sensitive issue and studies related to FSWs are lacking. Notably, in spite of the law against Malay prostitution, many FSWs in Malaysia were Malays (4). This may be attributable to their marriage problems and poor economic backgrounds. As pointed out by Hasan (20), divorce and poverty are the main reasons for Malay prostitution. Different from previous studies, the present study makes an additional effort to perform a comprehensive analysis by utilising an ordinal regression model to assess different levels of HIV knowledge. Information on sociodemographic variations in the likelihood of possessing different levels of HIV knowledge among FSWs would be of interest to policymakers and researchers from various disciplines.

## Methods

### Data

Cross-sectional data from the Integrated Biological and Behavioral Surveillance Survey (IBBS) 2017, that is, the most recent nationwide survey related to HIV in Malaysia, were used in secondary analysis (4). The survey covered all states in Malaysia and lasted from March 2017 to June 2017. The team members of the IBBS 2017 comprised medical assistants, nurses and community staff. The focus populations included FSWs. Given that FSWs were underserved populations, respondent-driven sampling (RDS) with a link-tracing network sampling strategy was used to collect data. RDS is an appropriate sampling approach for the hard-to-reach populations, especially when a clear sampling frame does not exist. At the initial stage of RDS, numerous initial respondents (seeds) were identified but only few were planted to commence the recruitment chain. If the recruitment was terminated, new seeds were planted.

Prior to interviewing the recruited respondents, thorough screening was conducted to determine eligibility, which was subject to the inclusion and exclusion criteria. The inclusion criteria were adult females who were 18 years old and above and were paid with money for sexual activity within the last 90 days. The exclusion criteria were those who could not understand the languages used by the interviewers. Each seed was given three coupons to gather the first wave of respondents from his/her network. Then, the recruited respondents were requested to recruit other respondents in the following wave until the targeted sample size was obtained. As an incentive, every respondent was given RM40 to participate in the survey and RM10 to recruit a new respondent.

Even though RDS is nonprobability sampling, the formula of simple random sampling was used to determine the targeted sample size. The calculation was based on the estimated population size of 40,000 FSWs, expected HIV prevalence of 4%, confidence limit of 3% and a design effect of 4. The calculated, targeted sample size was 808 FSWs, but only 630 were interviewed, which was equivalent to a response rate of approximately 78%. Nevertheless, this sample size was considered large and adequate for research, given that FSWs were hard-to-reach populations. The questionnaires were constructed according to the Family Health International Guidelines for Repeated Behavioural Surveys in Population at Risk of HIV and were prepared in two languages, i.e. the Malay and English languages. However, the interviewers were allowed to use other languages to conduct an interview as long as the respondents understood. During the face-to-face interview, the respondents were asked to report their sociodemographic profiles, sexual behaviours and HIV knowledge. Informed consent was obtained from the selected respondents prior to the interview. Ethical approval was sought from the National Research Committee of Malaysia.

### Outcome Variable

HIV knowledge, the outcome variable of the present study, was formatted as a categorical variable with three ordinal outcomes: i) low-, ii) middle- and iii) high-level HIV knowledge. This variable was formed based on five questions related to HIV asked in the questionnaire: i) “Can a person reduce the risk of HIV by having one faithful, uninfected sex partner?,” ii) “Can a person reduce the risk of HIV by using condom?,” iii) “Can a healthy-looking person have HIV?,” iv) “Can a person become infected through mosquito bites?,” and v) “Can a person get HIV by sharing meal with someone who is infected with HIV?” Each correct answer was given a value of 1 and each incorrect answer was given a value of 0. Therefore, 5 was the maximum value and 0 was the minimum value. Respondents with low-level HIV knowledge were those having scores of 0–2. Values of 3–4 and 5 referred to middle- and high-level HIV knowledge, respectively. Several published articles adopted a somewhat similar type of operationalisation of HIV knowledge for research (21–23). The reliability of constructing this outcome variable was assessed using Cronbach’s alpha. Its value was 0.62, which fell within the acceptable range (24). This indicated that the five HIV-related questions consistently measured HIV knowledge.

### Explanatory Variables

Considering the findings pertaining to HIV knowledge from previous empirical studies as well as data availability, the sociodemographic variables selected in the present study consisted of age, education, income, ethnicity and marital status (11, 12, 25–28). Information on sociodemographic profiles was self-reported by the respondents. The age of the respondents was segmented into four groups: i) ≤ 29 years old, ii) 30 years old–39 years old, iii) 40 years old–49 years old and iv) ≥ 50 years old. Respondents’ educational levels were stratified based on the local educational system: no formal education, primary, secondary and tertiary. The monthly individual income of the respondents was classified into five categories: i) ≤ RM499; ii) RM500–RM999; iii) RM1,000–RM1,499; iv) RM1,500–RM1,999 and v) ≥ RM2,000. Respondents’ ethnic backgrounds were categorised into four groups: i) Malay, ii) Chinese, iii) Indian and iv) other ethnicities (Others). The largest ethnic group in Malaysia is Malay, followed by Chinese, Indian and Others. All Malays are Muslims. Other ethnic groups comprised Iban, Kadazan, Dusun, Bajau and Bidayuh. The respondents were asked to report their marital status (i.e. single, married, divorced and widowed). Owing to the small number of observations, divorced was merged with widowed.

### Statistical Analyses

A total of 630 respondents were included in the current analyses. Prior to performing multivariate analyses, descriptive statistics of all variables were calculated. Then, Pearson’s chi-square tests of independence were used to assess the associations between HIV knowledge and sociodemographic variables. The number and proportion of respondents with different levels of HIV knowledge were calculated. Next, ordered probit regressions were utilised to evaluate the independent effects of sociodemographic variables on the probabilities of having low-, middle- and high-level HIV knowledge. The present study developed three regression models to identify whether the income, education and marital status variables contributed significantly to predicting HIV knowledge. This identification was necessary as it could help in determining the best model for the data. Model 1 consisted of age and ethnic variables. Education and income variables were added to Model 2 and Model 3 used all sociodemographic variables, including marital status. These three models were then compared based on their pseudo-R^2^ and Akaike’s information criterion (AIC). In addition, variance inflation factors (VIFs) were used to diagnose potential multicollinearities and link tests by Pregibon (29) were performed to evaluate model specification errors. The marginal effect and robust standard error of each explanatory variable in the best regression model were estimated and interpreted. The significance level of *P* < 0.05 was selected. Statistical analyses were performed using Stata statistical software (30).

## Results

Nearly half of the total respondents had middle-level HIV knowledge (44.1%), followed by those with high- (41%) and low-level knowledge (14.9%). Approximately 51.1% of the respondents aged ≤ 29 years old had high-level HIV knowledge, compared with 32.6% and 32.5% of those aged 40 years old–49 years old and ≥ 50 years old, respectively. Overall, more than half of the respondents had secondary-level education (54.6%) whereas only a very small proportion had tertiary-level education (3.5%). Low-level HIV knowledge was more prevalent among respondents without formal education (23.2%) compared with those with tertiary-level education (9.1%). Approximately one-third of the respondents had incomes of RM500– RM999 (34.3%); followed by those with incomes of RM1,000–RM1,499 (26.7%); RM1,500– RM1,999 (15.7%); ≤ RM499 (12.1%) and ≥ RM2,000 (11.3%). High-level HIV knowledge was most common among respondents with incomes of RM1,500–RM1,999 (63.6%), whereas low-level knowledge was most frequent in respondents having incomes of RM500–RM999 (19%). Large proportions of respondents were Malays (46%) and divorced/widowed (49.5%). Nearly 50% of the respondents of other ethnic groups had high-level HIV knowledge, compared with only 30%–33% of Chinese and Indians. A significantly lower proportion of single respondents had low-level HIV knowledge (12.8%) compared with divorced/widowed (13.1%) and married respondents (22.1%) (Table 1).

Comparing between regression models, the pseudo-R^2^ and AIC seemed to favour Model 3 over the other two models because it had the lowest AIC and the highest pseudo-R^2^. Furthermore, Model 3 did not have multicollinearities and specification errors as its maximum VIF was very low and prediction squared was insignificant. Collectively, the results of the present study indicated that income, education, and marital status were relevant variables and should not be omitted. The interpretation of the results was thereby based on Model 3 (Table 2).

Having tertiary-level education increased the probability of having high-level HIV knowledge by 19.5% and reduced the probability of having low-level HIV knowledge by 8.3%. Respondents with incomes of RM1,500–RM1,999 were 14.1% more likely to have high-level HIV knowledge compared to those having ≤ RM499. These respondents were also 6.8% less likely to have low-level HIV knowledge. Compared to married respondents, single respondents had an 8% lower probability of having low-level HIV knowledge and a 15.6% higher likelihood of having high-level HIV knowledge. Being divorced/widowed increased the probability of possessing high-level HIV knowledge by 10.2% and reduced the probability of having low-level HIV knowledge by 5.8% (Table 3).

## Discussion

The present study, using a large sample of FSWs in Malaysia and a strong analytical approach, explored the prevalence of HIV knowledge and its associated factors. A large proportion of FSWs was found to have high-level HIV knowledge and only a small number had low-level knowledge. Of all sociodemographic factors, only education, income and marital status were independently associated with FSWs’ HIV knowledge. However, age and ethnicity were not associated with HIV knowledge when other sociodemographic factors were controlled.

It is interesting and important to compare the prevalence of HIV knowledge obtained in the present study with the cases evidenced in other studies, even though the methodologies used by other studies were dissimilar. An early study, using a questionnaire containing 12 questions related to general knowledge of HIV infection, found that only 10% and 7.2% of brothel- and street-based sex workers in Bangladesh had a high number of correct responses to the questions, respectively (31). The study by Cai et al. (32) on HIV knowledge among FSWs in China found that more than half of FSWs (60.8%) provided correct answers for all items regarding HIV and up to 89.5% correctly answered that HIV could be transmitted through HIV-infected blood. Another study showed that 90.6% of FSWs in Peru never heard of HIV and nearly half (42.5%) believed that they faced a high risk of obtaining HIV (17). An empirical study on FSW in Rwanda across several years focusing on knowledge of HIV prevention methods found the percentage of FSWs with comprehensive HIV knowledge to increase from 18.4% in 2006 to 71.1% in 2015. Another study related to HIV behaviours in China pointed to the high awareness of HIV among FSWs (33). In particular, 91% of FSWs had heard of HIV, 84% believed that condoms could protect against HIV and 91% thought that sexual intercourse was a risk factor for HIV infection. In a similar study, Bruce et al. (34) revealed that a very large proportion of FSWs in Papua New Guinea had heard of HIV, knew that sexual activity could transmit HIV and was aware of the importance of condom use in HIV prevention. Sinha et al. (35), making use of the data from a cross-sectional study in India, observed that 90% of FSWs knew what HIV was. However, only a small proportion understood that HIV could be transmitted from infected syringe (32.2%). Yu et al. (36) assessed HIV knowledge among Vietnamese FSWs in China based on eight questions pertaining to HIV prevention measures. Assigning a value of 1 for each correct answer, the authors found the mean value to be 6.1, indicating that the majority of the FSWs had good HIV knowledge. Collectively, the findings from most previous studies were somewhat comparable to those of the present study, indicating that a large proportion of FSWs had basic HIV-related knowledge.

Well-educated FSWs in the present study had a higher likelihood of reporting high-level HIV knowledge and were less likely to report low-level HIV knowledge compared to those with a low educational background. This is perhaps because education improves health awareness and knowledge. Education could thus be seen as a potential protective factor for HIV transmission. Similar findings were evidenced in China (25, 28). In particular, Lau et al. (25), focusing on institutionalised FSWs in Shenzhen, suggested that FSWs with senior or junior high school qualifications tended to have better HIV/AIDS-related knowledge relative to their less educated peers who have attended primary schools only. In a later study that was based on three cross-sectional surveys, Liao et al. (28) used several questions to measure HIV knowledge and identified that FSWs were more knowledgeable if they were better educated. The results of other similar studies conducted elsewhere also showed a positive relationship between education and HIV knowledge (2, 12, 27). For instance, Kakchapati et al. (27) demonstrated that in Nepal, FSWs with secondary-level education or above were likely to have better awareness of using condoms to prevent HIV than their less educated counterparts, and Teshale et al. (2) found higher education to be associated with comprehensive HIV knowledge in the sub-Saharan African population. On the basis of the present study’s findings, suggested multifaceted intervention strategies could include education programmes to enhance HIV knowledge among the less educated segment of FSWs, that is, those without tertiary-level education. In these programmes, key information regarding the use of condoms and avoidance of needle sharing to prevent HIV should be well highlighted using various multilingual mass media (e.g. television, radio and newspaper) and social apps (e.g. Facebook, Twitter and Instagram). This is in light of the main findings from the study by Sakha et al. (12) that after an educational intervention, FSWs’ knowledge regarding sexually transmitted infections and the proportion of FSWs using condoms improved substantially.

The present study provided a new insight into the relationship between income and HIV knowledge. Although a few studies threw light on factors associated with HIV knowledge, the effect of income on HIV knowledge remained ambiguous (11, 12, 25–28). The inclusion of an income variable in the analysis is important in the sense that it can help in uncovering the influence of health value, which is measured by income, on HIV knowledge (37). Despite the paucity of research, there were two studies exploring the relationships between income and knowledge of health behaviours (i.e. active and passive smoking) among adults in Malaysia (38, 39). The authors of these studies found a negative relationship between income and knowledge, which was rather in contrast to the findings of the present study. In the present study, to certain extent, lower income FSWs were less likely to have high-level HIV knowledge and more likely to acquire low-level HIV knowledge when compared with their higher income counterparts. Somewhat similar findings were observed by Kareem et al. (40), who used a nationwide sample of Nigeria and found positive relationships between wealth quintiles and the odds of having comprehensive HIV knowledge. A plausible reason for this outcome is the high opportunity costs of being sick. As explained by Grossman (37), a health economist, if an individual gets sick, his or her opportunity to earn income will be forgone and income increases the value of health. Therefore, higher income FSWs could be more aware of their health than lower income FSWs. In terms of policy implication, although Malaysia has a high number of FSWs with high-level HIV knowledge in all income groups, policymakers and public health administrators could make concerted efforts to provide FSWs throughout the nation with insightful information on HIV. Special attention is suggested to be paid to FSWs in the lowest income group (i.e. ≤ RM499).

Findings pertaining to the association between marital status and HIV knowledge documented in previous studies were mixed. On one hand, using a sample collected in India, Halli et al. (26) found the odds of having good knowledge regarding HIV symptoms to be greater among married FSWs than the unmarried. On the other hand, in a study of 520 sex workers in Afghanistan, married FSWs were found to have poorer awareness of HIV compared with their unmarried peers because they had limited access to community-based HIV prevention programmes (11). However, the findings from the studies by Liao et al. (28) and Kakchapati et al. (27) showed no significant effect of marital status on HIV knowledge. These findings were unsurprising as FSWs often did not intend to declare themselves as married. The present study’s outcomes that being unmarried increased the likelihood of having high-level HIV knowledge seemed to be in agreement with those of Todd et al. (11), thereby concluding that marriage does not play an important role in improving HIV knowledge among FSWs. Considering the findings from the present study, nationwide intervention policies could be formulated to occur at a suitable time and in an appropriate setting in an effort to improve HIV knowledge in the FSW population with particular attention on the married FSWs. HIV-related health education campaigns could be the main element in these policies. To ensure that the campaigns reach a wider audience, health professionals from various ethnic backgrounds could be recruited as health educators.

In terms of bivariate analysis, age was associated with HIV knowledge. However, after controlling for other sociodemographic variables in the multiple regression, the relationship became insignificant, implying that FSWs’ age did not have an independent effect on their HIV knowledge. Findings from several previous studies suggested likewise, with age having no independent association with HIV knowledge (11, 26–28). It was also interesting to note that no independent relationship existed between ethnicity and HIV knowledge. This led to a conclusion that cultural and religious factors may not influence the tendency of FSWs to acquire HIV knowledge. An in-depth qualitative study is suggested to be carried out in the future to verify this conclusion and yield better knowledge of the influence of ethnicity on HIV awareness. Researchers can also devote their attention to exploring why Malays are more likely than non-Malays to engage in sex work, even though Syariah law is against Malay prostitution. Collectively, the present study’s findings imply that if resource constraints are a concern, public health administrators in the Ministry of Health Malaysia should not pay too much attention to age and ethnic factors when designing HIV-related interventionist strategies. Nevertheless, this does not mean that FSWs’ age and ethnic background can be ignored completely as there are minor age and ethnic variations in the prevalence of HIV knowledge.

Even though the present study offers comprehensive understanding of the relationships between sociodemographic factors and levels of HIV knowledge among FSWs, it has several unavoidable limitations. First, although the IBBS 2017 has a large sample size, it was conducted several years ago, and thus, it is unable to reflect the most up-to-date scenario in Malaysia. Second, cross-sectional data do not allow for causality analysis. As a result, the causal relationships between variables could not be well explored. Third, owing to the fact that sex work is prohibited by laws in Malaysia, it was difficult to reach FSWs throughout the nation. Lastly, reporting errors may occur because of self-reports of HIV knowledge by respondents. Despite these limitations, the present study is the first of its kind that conducts an in-depth examination of sociodemographic factors associated with HIV knowledge among FSWs in Malaysia. In addition, the rigorous statistical method and large data utilised in the present study generated important and useful findings. Moreover, the segments of the sex worker population that had strong or weak HIV knowledge were shed light. With the availability of longitudinal data, exploring the causal effect of HIV knowledge on HIV infection could be a direction for future empirical studies.

## Conclusion

Sociodemographic factors such as education, income and marital status play a major role in influencing the levels of HIV knowledge among FSWs. In particular, FSWs are more likely to have low-level HIV knowledge if they are less educated, lower income recipients and married. However, there appear to be no profound disparities in HIV knowledge levels across age and ethnic groups. The findings of the present study could provide public health policymakers with detailed information on which cohorts of the FSW population should be given special attention. Public health administrators are strongly recommended to take right measures to improve HIV knowledge among less knowledgeable FSWs identified in the present study. For instance, FSWs with poor educational backgrounds should be provided with adequate information regarding HIV through multilingual mass and social media. Special attention is paid not only to less educated FSWs but also to low-income FSWs. Furthermore, in nationwide HIV-related health education campaigns, health professionals of various ethnic groups can be invited to educate married FSWs regarding the risk of HIV. With these actions, the prevalence of HIV across the nation is expected to be reduced significantly.

## Figures and Tables

**Table 1:** Summary statistics of variables, by levels of knowledge of HIV

Variables	Total (*N* = 630)	Knowledge of HIV			*P*-value

		Low (*n* = 94)	Middle (*n* = 278)	High (*n* = 258)	
Age (years old)					
≤ 29	186 (29.5)	29 (15.6)	62 (33.3)	95 (51.1)	< 0.004
30–39	183 (29.1)	27 (14.8)	78 (42.6)	78 (42.6)	
40–49	138 (21.9)	19 (13.8)	74 (53.6)	45 (32.6)	
≥ 50	123 (19.5)	19 (15.5)	64 (52.0)	40 (32.5)	
Education					
No formal	95 (15.1)	22 (23.2)	32 (33.7)	41(43.2)	0.002
Primary	169 (26.8)	23 (13.6)	91 (53.9)	55 (32.5)	
Secondary	344 (54.6)	47 (13.7)	150 (43.6)	147 (42.7)	
Tertiary	22 (3.5)	2 (9.1)	5 (22.7)	15 (68.2)	
Income (RM)					
≤ 499	76 (12.1)	12 (15.8)	30 (39.5)	34 (44.7)	< 0.001
500–999	216 (34.3)	41 (19.0)	105 (48.6)	70 (32.4)	
1,000–1,499	168 (26.7)	24 (14.3)	89 (53.0)	55 (32.7)	
1,500–1,999	99 (15.7)	8 (8.1)	28 (28.3)	63 (63.6)	
≥ 2,000	71 (11.3)	9 (12.7)	26 (36.6)	36 (50.7)	
Ethnicity					
Malay	290 (46.0)	39 (13.5)	128 (44.1)	123 (42.4)	0.008
Chinese	69 (11.0)	7 (10.1)	39 (56.5)	23 (33.3)	
Indian	90 (14.3)	14 (15.6)	49 (54.4)	27 (30.0)	
Others	181 (28.7)	34 (18.8)	62 (34.3)	85 (47.0)	
Marital status					
Married	131 (20.8)	29 (22.1)	63 (48.1)	39 (29.8)	0.001
Single	187 (29.7)	24 (12.8)	67 (35.8)	96 (51.3)	
Divorced/widowed	312 (49.5)	41 (13.1)	148 (47.4)	123 (39.4)	

Source: IBBS 2017

Note: The entries refer to counts; For total sample, column percentages are shown in parentheses; For respondents with low-, middle- and high-level HIV knowledge, row percentages are shown in parentheses; *P*-values for Pearson’s chi-squared (χ^2^) tests of independence; The significance level is *P* < 0.05

**Table 2:** Estimated coefficients for the multivariable regression models (*N* = 630)

Variables	Ordered probit		

	Model 1	Model 2	Model 3
Age (years old)			
≤ 29	Ref.	Ref.	Ref.
30–39	−0.139 (0.129)	−0.054 (0.132)	0.013 (0.139)
40–49	−0.276* (0.130)	−0.114 (0.133)	−0.060 (0.145)
≥ 50	−0.294* (0.142)	−0.114 (0.154)	−0.075 (0.168)
Education			
No formal	−	Ref.	Ref.
Primary	−	0.018 (0.157)	0.051 (0.158)
Secondary	−	0.150 (0.151)	0.178 (0.154)
Tertiary	−	0.507* (0.312)	0.493* (0.306)
Income (RM)			
≤ 499	−	Ref.	Ref.
500–999	−	−0.278 (0.158)	−0.262 (0.159)
1,000–1,499	−	−0.216 (0.164)	−0.236 (0.166)
1,500–1,999	−	0.390* (0.194)	0.357* (0.195)
≥ 2,000	−	0.090 (0.204)	0.084 (0.205)
Ethnicity			
Malay	Ref.	Ref.	Ref.
Chinese	−0.018 (0.141)	0.030 (0.145)	0.036 (0.146)
Indian	−0.176 (0.126)	−0.103 (0.131)	−0.065 (0.134)
Others	−0.063 (0.117)	0.002 (0.120)	−0.003 (0.121)
Marital status			
Married	−	−	Ref.
Single	−	−	0.398* (0.142)
Divorced/widowed	−	−	0.262* (0.122)
Wald test	8.630	39.760	47.170
*P*-value	0.196	< 0.001	< 0.001
Prediction squared	−1.352	0.955	0.602
*P*-value	0.563	0.058	0.098
Maximum VIF	1.590	2.590	2.780
Pseudo R^2^	0.007	0.031	0.037
AIC	1279.920	1264.412	1259.729

Source: IBBS 2017

Note: VIF refers to variance inflation factor. AIC refers to Akaike’s information criterion. Robust standard errors in parentheses. The significance level is *P* < 0.05;

* *P* < 0.05

**Table 3:** Estimated marginal effects for the ordered probit model (*N* = 630)

Variables	Knowledge of HIV		

	Low	Middle	High
Age (years old)			
≤ 29	Ref.	Ref.	Ref.
30–39	−0.003 (0.030)	−0.002 (0.024)	0.005 (0.054)
40–49	0.014 (0.033)	0.010 (0.023)	−0.023 (0.056)
≥ 50	0.017 (0.039)	0.012 (0.026)	−0.029 (0.064)
Education			
No formal	Ref.	Ref.	Ref.
Primary	−0.011 (0.034)	−0.009 (0.028)	0.020 (0.062)
Secondary	−0.039 (0.035)	−0.029 (0.025)	0.069 (0.059)
Tertiary	−0.083* (0.038)	−0.112 (0.082)	0.195* (0.118)
Income (RM)			
≤ 499	Ref.	Ref.	Ref.
500–999	0.061 (0.038)	0.041 (0.023)	−0.101 (0.060)
1,000–1,499	0.055 (0.041)	0.035 (0.022)	−0.090 (0.062)
1,500–1,999	−0.068* (0.032)	−0.073 (0.046)	0.141* (0.077)
≥ 2,000	−0.018 (0.042)	−0.015 (0.039)	0.033 (0.080)
Ethnicity			
Malay	Ref.	Ref.	Ref.
Chinese	−0.008 (0.031)	−0.006 (0.026)	0.014 (0.057)
Indian	0.015 (0.031)	0.011 (0.020)	−0.025 (0.051)
Others	0.001 (0.027)	0.001 (0.020)	−0.001 (0.047)
Marital status			
Married	Ref.	Ref.	Ref.
Single	−0.080* (0.026)	−0.076* (0.031)	0.156* (0.055)
Divorced/widowed	−0.058* (0.027)	−0.044* (0.021)	0.102* (0.047)

Source: IBBS 2017

Note: Robust standard errors in parentheses. The significance level is *P* < 0.05;

* *P* < 0.05
